# Genotypic and Phenotypic Assessment of Hyaluronidase among Type Strains of a Select Group of Staphylococcal Species

**DOI:** 10.1155/2009/614371

**Published:** 2010-01-10

**Authors:** Mark E. Hart, Morgan J. Hart, Anna J. Roop

**Affiliations:** ^1^Division of Microbiology, National Center for Toxicological Research, U.S. Food and Drug Administration, Jefferson, AR 72079, USA; ^2^Department of Microbiology and Immunology, University of Arkansas for Medical Sciences, Little Rock, AR 72205, USA; ^3^Department of Biology, Ouachita Baptist University, Arkadelphia, AR 71998, USA

## Abstract

Hyaluronidases degrade hyaluronic acid, a major polysaccharide of the extracellular matrix of tissues, and are considered important for virulence in a number of Gram-positive and -negative bacteria. The purpose of the present study was to determine the prevalence of hyaluronidase among clinical strains of *Staphylococcus aureus* and among other *Staphylococcus* species. Spent media and chromosomal DNA were assessed for hyaluronidase activity and the absence or presence of a hyaluronidase gene (*hysA*) by Southern analysis, respectively. All *S. aureus* strains examined exhibited at least one hybridizing band (half of the strains exhibited two or more hybridizing bands) when probed for *hysA* and all but three of these strains produced hyaluronidase. In contrast, none of the type strains of 19 other species exhibited either hyaluronidase activity or hybridizing bands when probed for *hysA*. These data support the hypothesis that among members of the *Staphylococcus* genus only strains of *S. aureus* possess the enzyme hyaluronidase. This would suggest that hyaluronidase represents yet another potential virulence factor employed by *S. aureus* to cause disease and may represent a diagnostically important characteristic for distinguishing *S. aureus* from other members of this genus.

## 1. Introduction

Hyaluronidase, an enzyme that primarily degrades hyaluronic acid, a major component of the extracellular matrix of human tissues, has been found in both Gram-positive and -negative bacteria and, in several cases, has been implicated in the disease process as a means to either invade tissues or generate a source of carbon and energy [1–8]. A role for hyaluronidase in *S. aureus* diseases has been suggested by Makris et al. [9], who demonstrated, using a mouse abscess model, that the number of viable cells recovered for a hyaluronidase mutant strain was significantly less than that for its parent strain. Recent proteomic studies of spent media from *Staphylococcus aureus* UAMS-1 and its regulatory mutants, *sarA*, *agr*, and *sarA agr*, detected two different proteins identified as hyaluronidases [10]. Concentrations of these proteins were higher in the *sarA* and *sarA agr* mutant strains than in the parent and *agr* mutant strains, suggesting the involvement of SarA, an important regulator of virulence in *S. aureus*, in the regulation of hyaluronidase [10]. While the exact role of hyaluronidase in *S. aureus* is not known, the involvement of *sarA* in the regulation of the hyaluronidase gene (*hysA*) expression [9, 10] and the identification of genes annotated as putative hyaluronidases in all fourteen sequenced strains of *S. aureus* [11] would indicate that *S. aureus* hyaluronidase is a virulence factor important for some aspect of disease. 

The genus *Staphylococcus* is a diverse group of some 41 known species [12, 13]. In the human clinical setting the production of coagulase is usually used to distinguish between *S. aureus* and all other species, which are typically grouped together as the coagulase-negative staphylococci (CoNS) [14]. However, species other than *S. aureus* produce coagulase even though their encounter in the human clinical setting is rare [14–18]. These species are *S. intermedius*, *S. delphini*, *S. schleiferi* subsp. *coagulans*, *S. hyicus *subsp*. hyicus*, and *S. lutrae* [13, 19–22]. 

The presence or absence of hyaluronidase among the staphylococci has been examined, previously. For example, Choudhuri and Chakrabarty [23] examined 523 staphylococcal isolates from human pathologies, and of the 96% that were coagulase positive, only 13% were negative for hyaluronidase activity. Nine of the remaining 21 strains that were coagulase-negative were shown not to produce hyaluronidase [23]. Essers and Radebold [24] examined 368 staphylococcal strains isolated from human specimens, and of the 218 determined to be coagulase positive, only one isolate was hyaluronidase negative. Of the remaining 150 strains determined to be coagulase negative, only one isolate had hyaluronidase activity [24]. An additional 495 strains were examined for their ability to produce DNAse and hyaluronidase [24]. Only ten of the 323 DNAse positive strains were negative for hyaluronidase activity while 170 of the remaining 172 strains designated DNAse negative were also negative for hyaluronidase activity. Collectively, these data [23, 24] indicate that hyaluronidase activity is predominantly seen among strains of staphylococci that exhibit both coagulase and DNAse activity, two characteristics typically used to distinguish *S. aureus* from other staphylococcal species, at least in the context of human disease [13]. While it appears that hyaluronidase activity is found among nearly all strains of *S. aureus*, *S. hyicus* subsp. *hyicus*, a strain known to cause infectious exudative epidermititis in swine, has also been reported positive for hyaluronidase activity [13, 19, 25]. To the best of our knowledge, hyaluronidase activity among other species of *Staphylococcus* has not been systematically examined. The purpose of the present study was to determine the prevalence of hyaluronidase in members of the genus *Staphylococcus*, primarily those associated with human disease.

## 2. Materials and Methods

### 2.1. Staphylococcal Strains and Growth Conditions

*S. aureus* strains used in this study are listed in Table 1. While these strains were obtained primarily from two sources, the University of Arkansas for Medical Sciences and the University of Nebraska Medical Center, the strains represent a diverse group of clinical isolates originally obtained from different geographical locations, outbreaks, and disease syndromes. Other species examined were *S. auricularis* ATCC 33753^**T**^, *S. capitis* subsp. *capitis* ATCC 27840^**T**^, *S. caprae* ATCC 35538^**T**^, *S. carnosus* subsp. *carnosus* ATCC 51365^**T**^, *S. chromogenes *ATCC 43764^**T**^, *S. cohnii* subsp. *cohnii* ATCC 29974^**T**^, *S. delphini* ATCC 49171^**T**^, *S. epidermidis* ATCC 14990^**T**^, *S. epidermidis* ATCC 12228, *S. haemolyticus* ATCC 29970^**T**^, *S. hominis *subsp. *hominis* ATCC 27844^**T**^, *S. hyicus* subsp. *hyicus* ATCC 11249^**T**^, *S. intermedius* ATCC 29663^**T**^, *S. lugdunensis* ATCC 43809^**T**^, *S. saprophyticus* ATCC 19701^**T**^, *S. schleiferi* subsp. *coagulans* ATCC 49549^**T**^, *S. schleiferi* subsp. *schleiferi* ATCC 43808^**T**^, *S. sciuri* subsp. *sciuri* ATCC 29062^**T**^, *S. simulans* ATCC 27848^**T**^, *S. warneri* ATCC 27836^**T**^, and *S. xylosus* ATCC 29971^**T**^. Strains were maintained as frozen (−80°C) stocks in brain heart infusion broth (BHI; Difco Laboratories, Detroit, Mich.) containing 25% (w/v) glycerol and routinely streaked for isolation on tryptic soy broth (TSB; Difco) containing agar (1.5%). Flasks containing 20 mL of TSB were inoculated from plate cultures and incubated at 37°C overnight (15–18 hours) with rotary (180 rpm) aeration.

### 2.2. DNA Isolation and Southern Analysis

Chromosomal DNA was isolated using the GenElute Bacterial Genomic DNA Kit (Sigma-Aldrich Chemical Co., St. Louis, Mo) and digested with *Cla*I restriction endonuclease, resolved by agarose gel electrophoresis, and transferred to neutral nylon membranes (MagnaGraph; Micron Separations Inc., Westborough, Mass.) by passive diffusion. Membranes were hybridized overnight with a digoxigenin-labeled (Roche Molecular Biochemicals, Indianapolis, Ind.) amplicon generated by PCR using either *S. aureus* UAMS-1 or RN6390 (8325 lineage) chromosomal DNA as template and primers (5′-GTGGATTGTTTGACAGTAGACAG-3′ and 5′-CGGTATTTGTAGATTCGGGATTATAG-3′) designed from the known genomic sequence of *S. aureus* N315 [26]. The amplicons were cloned and their identities were verified by sequencing. Each amplicon (probe) was 2,442 bp in size beginning 116 bases upstream of the start codon and ending approximately 100 bases short of the end of the gene. 

Hybridization was carried out at 65°C and hybridizing bands were detected by autoradiography using alkaline phosphatase-conjugated antidigoxigenin F(ab′)_2_ antibody fragments (Roche Molecular Biochemicals) and the chemiluminescent substrate CDP-*Star* (Roche Molecular Biochemicals).

### 2.3. Concentration of Spent Media

Optical density readings of overnight cultures (15–18 hours) were determined spectrophotometrically at 550 nm and used to dilute each culture with TSB to an optical density of 3.0 in a total volume of 10 mL. Diluted cultures were centrifuged (10,000 ×*g*, for 10 minutes at 4°C) and spent media were filter sterilized before concentration using ultrafiltration (Centricon Ultracel YM-3 filters with 3,000 MWCO, Millipore Corporation, Bedford, Maine) and a one-hour centrifugation (7,500 ×*g*) at 4°C. The retentates were recovered and stored at −80°C until needed.

### 2.4. Plate Assay for Detecting Hyaluronidase in Spent Media

Sterile plastic square plates containing 1% (w/v) SeaKem LE agarose (Cambrex BioScience Rockland, Inc., Rockland, Maine), 1% (w/v) bovine serum albumin (BSA; Fraction V; Fisher Scientific, Fair Lawn, NJ), and 0.4 mg mL^−1^ of hyaluronic acid (HA; Sigma, H-1504; potassium salt, from human umbilical cord) in 0.3 M sodium phosphate buffer (pH 5.3) were prepared according to Steiner and Cruce [27]. Once the agarose medium containing BSA and HA had solidified, 4 mm wells were made aseptically, and 20 *μ*L of spent media was pipetted into each well. Plates were incubated overnight (15–18 hours) at 37°C prior to flooding each plate with 2 M acetic acid. Clear zones were visualized against a background of opaque precipitated BSA conjugated to undigested HA and their diameters measured in millimeters. Purified bovine testicular hyaluronidase (Sigma, H-3506) was used as a positive control.

### 2.5. Plate Assay for Detecting Hyaluronidase-Producing Colonies in Mixed Culture

Tryptic soy broth (6 g in 120 mL of water) containing 1% (w/v) agarose was sterilized by autoclaving. The molten medium was equilibrated to 50°C prior to the addition of 40 mL of filter-sterilized BSA (5% [w/v] prepared in water) and 40 mL of filter-sterilized HA (1 mg mL^−1^ prepared in water) both equilibrated to 50°C. The suspension was mixed and dispensed as 20 mL portions into each of ten Petri plates (15 × 100 mm). The medium (TSHA) was allowed to solidify and plates were incubated overnight at 37°C before use. Overnight cultures of *S. aureus* strains ATCC 33753 and Newman and *S. epidermidis* strain ATCC 12228 were diluted with TSB, mixed in equal portions, and plated onto TSHA plates to yield 30-300 colonies per plate. Plates were incubated overnight (15–18 hours) at 37°C prior to flooding each plate with 2 M acetic acid to precipitate undigested HA conjugated to BSA.

## 3. Results

### 3.1. Hyaluronidase Activity of *S. Aureus*

Hyaluronidase activity was detected for 93% (40 of 43) of *S. aureus* strains (Table 1) examined while none of the remaining species exhibited hyaluronidase activity (data not shown) including *S. hyicus* subsp. *hyicus* ATCC 11249^**T**^ (Figure 3(a)); the only species other than *S. aureus* reported to have hyaluronidase activity [13, 19, 25]. 

The hyaluronidase-producing strains of *S. aureus* examined included three ATCC strains, eight sequenced strains, and two isolates of pulse-field type, USA-300 (Miss and LAC) (Table 1). The remaining strains examined included 17 vaginal toxic shock syndrome- (TSS-) producing clinical isolates and 15 clinical strains (Table 1). The identity of the three hyaluronidase nonproducing strains as *S. aureus* (NCH 241, 242, and 250) was determined by Gram stain, growth on mannitol salt agar and catalase, coagulase, and DNAse activities (data not shown). Their identities were confirmed using the Vitek 2 Compact (bioMérieux, Durham, N.C.) automated system and the ID-GP identification cards (bioMérieux). Strain NCH 242 was identified as *S. aureus* with low discrimination between it and *S. intermedius* but was positive with the Voges-Proskauer test. *Staphylococcus intermedius*, like *S. aureus*, produces coagulase and DNAse as well as utilize mannitol [13]. However, unlike *S. aureus*, S. *intermedius* is unable to ferment glucose to acetylmethylcarbinol (acetoin), and thus, is negative with the Voges-Proskauer test [13].

### 3.2. Southern Analysis

Chromosomal DNA was isolated from all strains and digested with the restriction endonuclease, *Cla*I. This enzyme was chosen because it does not cut within the open reading frame of any of the hyaluronidase genes found in the 14 sequenced *S. aureus* genomic databases (data not shown). Digested DNA resolved by gel electrophoresis was analyzed by Southern analysis using a probe generated from the sequence of one of the hyaluronidase genes (*hysA1*) found in *S. aureus* UAMS-1 and from the corresponding gene found in *S. aureus* RN6390. Under high stringency conditions (65°C and low salt) both probes hybridized to the same *Cla*I fragment containing a hyaluronidase gene with equal intensities (data not shown). *Staphylococcus aureus* SH1000 (8325 lineage) and Sanger-252 were included in all Southern analysis as representative strains containing one and two *hysA* genes, respectively, as demonstrated by sequence analysis of their respective databases and independent cloning and sequencing of all three genes (data not shown). 

**Figure 1:**
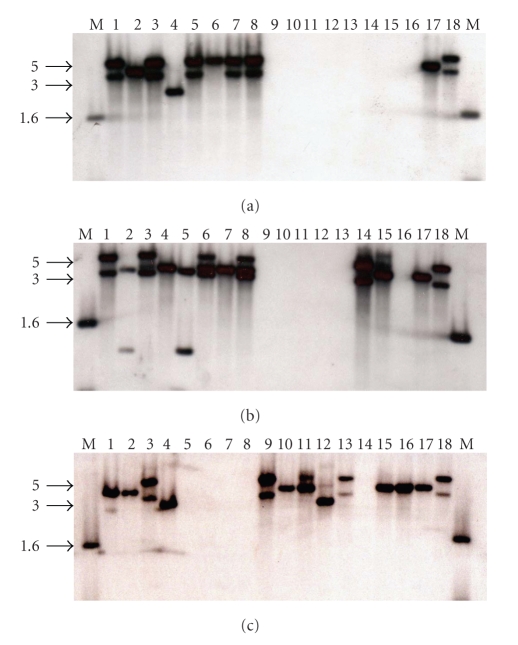
Southern analysis of chromosomal DNA from *Staphylococcus* species hybridized with a probe specific for *hysA*. Panel (a) *S. aureus* NCH 239 (1), NCH 240 (2), NCH 241 (3), NCH 242 (4), NCH 243 (5), NCH 244 (6), NCH 245 (7), NCH 246 (8), *S. auricularis* ATCC 33753^**T**^ (9), *S. capitis* subsp. *capitis* ATCC 27840^**T**^ (10), *S. caprae* ATCC 35538^**T**^ (11), *S. carnosus* subsp. *carnosus* ATCC 51365^**T**^ (12), S. *chromogenes* ATCC 43764^**T**^ (13), *S. cohnii* subsp. *cohnii* ATCC 29974^**T**^ (14), S. *delphini* ATCC 49171^**T**^ (15), *S. hominis* subsp. *hominis* ATCC 27844^**T**^ (16), *S. aureus* NCH 78 (17), and NCH 88 (18). Panel (b) *S. aureus* NTH 74 (1), NTH 75 (2), NTH 76 (3), NTH 77 (4), NTH 78 (5), NTH 79 (6), NTH 80 (7), NTH 81 (8), *S. intermedius* ATCC 29663^**T**^ (9), *S. lugdunensis* ATCC 43809^**T**^ (10), *S. saprophyticus* ATCC 19701^**T**^ (11), S. *schleiferi* subsp. *schleiferi* ATCC 43808^**T**^ (12), *S. epidermidis* ATCC 12228 (13), *S. aureus* NTH 125 (14), NCH 265 (15), *S. intermedius* ATCC 29633^**T**^ (16), NCH 78 (17), and NCH 88 (18). Panel (c) *S. aureus* NCH 47 (1), NCH 48 (2), NCH 49 (3), NCH 51 (4), *S. sciuri* subsp. *sciuri* ATCC 29062^**T**^ (5), *S. simulans* ATCC 27848^**T**^ (6), *S. warneri* ATCC 27836^**T**^ (7), *S. xylosus* ATCC 29971^**T**^ (8), *S. aureus* NTH 13 (9), NCH 78 (10), NCH 79 (11), NTH 82 (12), NCH 88 (13), *S. haemolyticus* ATCC 29970^**T**^ (14), NCH 328 (15), NCH 331 (16), NCH 78 (17), and NCH 88 (18). M: marker (kilobases).

**Figure 2:**
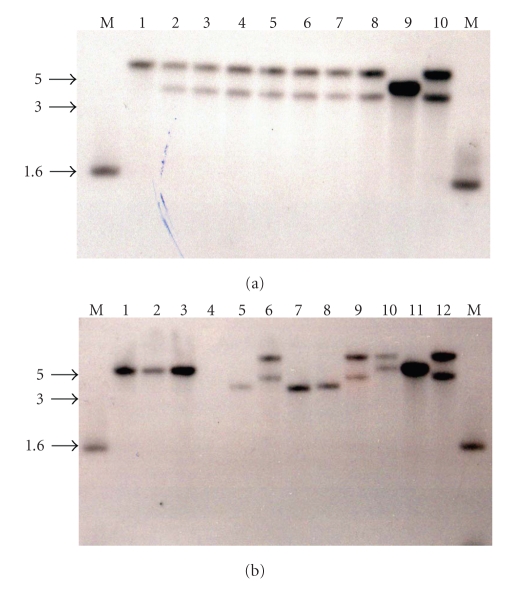
Southern analysis of chromosomal DNA from *Staphylococcus* species hybridized with a probe specific for *hysA*. Panel (a) *S. aureus* NCH 247 (1), NCH 248 (2), NCH 249 (3), NCH 250 (4), NCH 251 (5), NCH 252 (6), NCH 253 (7), NCH 254 (8), NCH 78 (9), and NCH 88 (10). Panel (b) *S. aureus* NCH 310 (1), NCH 338 (2), NCH 340 (3), *S. epidermidis* ATCC 14990^**T**^ (4), *S. aureus* NTH 68 (5), NTH 69 (6), NTH 70 (7), NTH 71 (8), NTH 72 (9), NTH 73 (10), NCH 78 (11), and NCH 88 (12). M: marker (kilobases).

**Figure 3:**
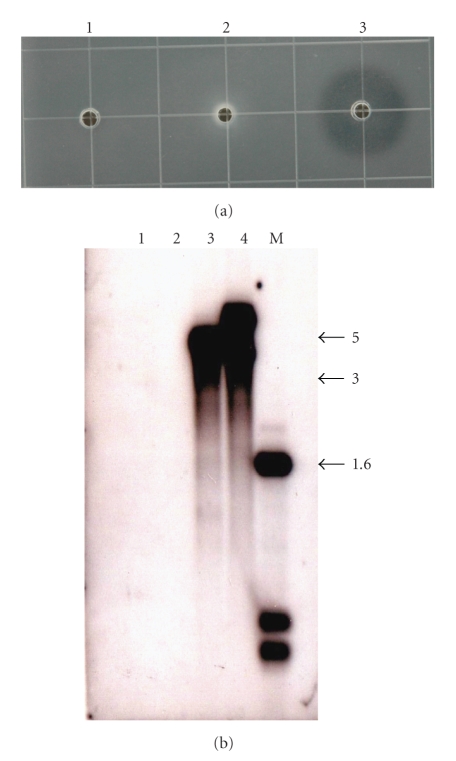
Panel (a) Hyaluronidase activity from spent media isolated from *S. hyicus* subsp. *hyicus* ATCC 11249^**T**^ (1), *S. schleiferi* subsp. *coagulans* ATCC 49545^**T**^ (2), and bovine testicular hyaluronidase (3). Panel (b) Southern analysis of *Cla*I-digested chromosomal DNA isolated from *S. hyicus* subsp. *hyicus* ATCC 11249^**T**^ (1), S. *schleiferi* subsp. *coagulans* ATCC 49545^**T**^ (2), *S. aureus* SH1000 (3), and Sanger-252 (4). M: marker (kilobases).

Approximately half (53.5%) of the 43 *S. aureus* strains examined exhibited two hybridizing bands and two strains (NTH 79 and 81) exhibited three hybridizing bands (Table 1 and Figures 1 and 2). These data suggest that these strains contain multiple copies of the hyaluronidase gene. The existence of smaller *Cla*I hybridizing fragments was noted for two strains, NTH 75 and NTH 78, and so the potential for restriction fragment length polymorphism exists. None of the remaining 19 species of *Staphylococcus* exhibited a hybridizing band for *hysA* (Figures 1 and 2) including the type strain for *S. hyicus* subsp. *hyicus* ATCC 11249^**T**^ (Figure 3(b)).

### 3.3. Detecting Hyaluronidase-Producing Strains on TSHA

**Figure 4:**
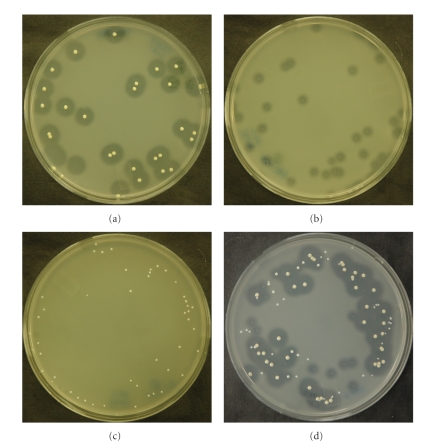
Hyaluronidase detection on tryptic soy agar supplemented with hyaluronic acid. *S. aureus* ATCC 33753 (a), *S. aureus* Newman (b), *S. epidermidis* ATCC 12228 (c), and a mixture of all three (d). Colonies of *S. aureus* Newman did not adhere to the agar surface when acetic acid was removed from the plates for photographic documentation.

Three strains of *Staphylococcus*, *S. aureus* strain ATCC 33753, *S. aureus* strain Newman, and *S. epidermidis* strain ATCC 12228 exhibiting a large (13.2 ± 1.3), small (5.8 ± 2.5), and no zone of activity on HA plates, respectively, were mixed and plated onto TSHA to determine whether or not TSHA could be used to discern *Staphylococcus* strains expressing hyaluronidase at different levels. All three strains could easily be discerned, as evident by the differences in zone sizes after overnight incubation at 37°C (Figure 4).

## 4. Discussion

Previous studies [23, 24] examined a large number of staphylococcal strains for the presence of hyaluronidase activity. While species identity was not determined in most cases, the strains were characterized as either positive or negative for coagulase or DNAse activities, characteristics typically used to distinguish *S. aureus* from other staphylococcal species [13]. In these studies [23, 24] the vast majority (93%) of strains that exhibited coagulase and DNAse activities also exhibited hyaluronidase activity. 

In the present study, we have examined the type strain for 20 different species of *Staphylococcus* for their ability to degrade hyaluronic acid and of these, only *S. aureus* strains exhibited hyaluronidase activity. In addition, over half of the *S. aureus* strains examined exhibited two or more genes for hyaluronidase. While the number of genes per strain will need to be confirmed, we know from the genomic databases of *S. aureus* Sanger-252 and RF-122 [11, 28, 29] and the proteomic analysis of *S. aureus* UAMS-1 [10] that these strains contain two genes for hyaluronidase. Whether or not the presence of two or more hyaluronidase genes affords some metabolic or pathogenic advantage is currently under investigation. However, it must be pointed out that an increase in the number of potential genes for any one strain did not necessarily result in an increase in hyaluronidase activity as determined with the hyaluronidase assay used in this study. In addition, not all *S. aureus* strains produced hyaluronidase although Southern analysis indicated the presence of at least one gene in each of the three strains that failed to exhibit hyaluronidase activity. It is also interesting that two of these hyaluronidase nonproducing strains appear to have two *hysA* genes. While at present we have no supporting data, this might suggest that either these strains carry two copies of the same defective gene or an important regulator of *hysA* expression is nonfunctional. Nevertheless, given the limited number of *S. aureus* strains examined in our study and the fact that some strains did not produce hyaluronidase would suggest that the lack of hyaluronidase by *S. aureus* is more prevalent than originally thought and that the enzyme may not be a contributing factor in virulence. Whether this is true or not will require further investigation particularly given that hyaluronidase has been implicated as a virulence factor in the mouse abscess model [9] and that the primarily virulence gene regulator, SarA, controls its expression [9, 10]. 

We are also intrigued that under our assay conditions as well as the Southern analysis that *S. hyicus* subsp. *hyicus*, a strain known to cause infectious exudative epidermititis in swine and reported to have hyaluronidase activity [13, 19, 25], exhibited no activity nor did it have a hybridizing band corresponding to the *hysA* gene from *S. aureus*. Currently, it is unclear as to why this discrepancy exists other than previous investigations [13, 19, 25] relied on the appearance of nonmucoid colonies of *Streptococcus equi*, which possesses a hyaluronic acid-containing capsule [30, 31] on blood agar when *Streptococcus equi* was plated as a lawn and *Staphylococcus* species, including *S. hyicus* were “spot” inoculated onto the lawn [32]. This would suggest, perhaps, that some factor other than hyaluronidase contributes to the formation of nonmucoid *Streptococcus equi *colonies. 

Lastly, while the method employed in this study clearly distinguished hyaluronidase-producing colonies from those that did not, more than likely the detection of hyaluronidase activity as a diagnostic test for the presumptive identification of *S. aureus* would not be feasible given the fact that not all *S. aureus* strains possess hyaluronidase activity. However, using methods that detect the presence or absence of the gene could possibly be used as 100% of the *S. aureus* strains examined in this study contained at least one gene for *hysA*.

## Figures and Tables

**Table 1:** Hyaluronidase activity of *Staphylococcus aureus *strains.

Strain	Relevant description	Source	HA activity*	*hysA* ^#^
NCH 265	ATCC 29213	ATCC^§^	13.2 ± 1.3	1
NCH 340	ATCC 12600^**T**^	ATCC	9.7 ± 0.6	1
NTH 125	ATCC 25923	ATCC	8.7 ± 0.8	2
NCH 49	MN8 (vaginal TSS**^¶^**)	NARSA^†^	8.7 ± 0.3	2
NCH 47	Mu50 (HA-MRSA)	NARSA	9.2 ± 0.3	1
NCH 51	MW2 (CA-MRSA)	NARSA	9.5 ± 0.5	1
NCH 48	N315 (HA-MRSA)	NARSA	7.0 ± 0.5	1
NCH 328	Newman	T. Foster, Trinity College, Dublin, Ireland	5.8 ± 2.5	1
NCH 331	Newman (*sigB*)	T. Foster, Trinity College, Dublin, Ireland	10.0 ± 0	1
NCH 88	Sanger-252 (EMRSA-16)	NARSA	7.2 ± 2.0	2
NCH 78	SH1000 (RN6390, *rsb**^+^***)	S. Foster, Univ. Sheffield, Sheffield, England	11.3 ± 0.6	1
NCH 79	SH1000 (RN6390, *rsb*)	S. Foster, Univ. Sheffield, Sheffield, England	12.5 ± 3.0	1
NTH 13	UAMS-1	M.S. Smeltzer, UAMS^¦^	9.0 ± 1.3	2
NCH 310	USA300 (Miss.)	M.S. Smeltzer, UAMS	9.8 ± 0.3	1
NCH 338	USA300 (LAC)	A.R. Horswill, University of Iowa	10.2 ± 1.6	1
NCH 239	Vaginal TSS	P.D. Fey, UNMC**^!^**	6.8 ± 0.6	2
NCH 240	Vaginal TSS	P.D. Fey, UNMC	8.8 ± 1.8	1
NCH 241	Vaginal TSS	P.D. Fey, UNMC	0 ± 0	2
NCH 242	Vaginal TSS	P.D. Fey, UNMC	0 ± 0	1
NCH 243	Vaginal TSS	P.D. Fey, UNMC	8.5 ± 0.7	2
NCH 244	Vaginal TSS	P.D. Fey, UNMC	10.0 ± 0.5	1
NCH 245	Vaginal TSS	P.D. Fey, UNMC	9.7 ± 0.6	2
NCH 246	Vaginal TSS	P.D. Fey, UNMC	7.8 ± 0.8	2
NCH 247	Vaginal TSS	P.D. Fey, UNMC	6.7 ± 1.2	1
NCH 248	Vaginal TSS	P.D. Fey, UNMC	8.0 ± 1.7	2
NCH 249	Vaginal TSS	P.D. Fey, UNMC	8.0 ± 0.9	2
NCH 250	Vaginal TSS	P.D. Fey, UNMC	0 ± 0	2
NCH 251	Vaginal TSS	P.D. Fey, UNMC	8.3 ± 1.0	2
NCH 252	Vaginal TSS	P.D. Fey, UNMC	7.3 ± 0.8	2
NCH 253	Vaginal TSS	P.D. Fey, UNMC	6.8 ± 1.0	2
NCH 254	Vaginal TSS	P.D. Fey, UNMC	8.2 ± 0.8	2
NTH 68	UAMS-625 (Abscess)	M.S. Smeltzer, UAMS	11.0 ± 3.0	1
NTH 69	UAMS-632 (Wound)	M.S. Smeltzer, UAMS	6.2 ± 1.0	2
NTH 70	UAMS-635 (Blood)	M.S. Smeltzer, UAMS	13.5 ± 1.3	1
NTH 71	UAMS-636 (Wound)	M.S. Smeltzer, UAMS	13.0 ± 0	1
NTH 72	UAMS-639 (Tracheal)	M.S. Smeltzer, UAMS	8.7 ± 0.8	2
NTH 73	UAMS-640 (Abscess)	M.S. Smeltzer, UAMS	10.3 ± 0.6	2
NTH 74	UAMS-641 (Blood)	M.S. Smeltzer, UAMS	7.2 ± 1.0	2
NTH 75	UAMS-655 (Blood)	M.S. Smeltzer, UAMS	13.2 ± 0.8	2
NTH 76	UAMS-682 (Outbreak 2)	M.S. Smeltzer, UAMS	12.0 ± 1.0	2
NTH 77	UAMS-687	M.S. Smeltzer, UAMS	9.0 ± 0.5	1
NTH 78	UAMS-688	M.S. Smeltzer, UAMS	11.8 ± 0.8	2
NTH 79	UAMS-689	M.S. Smeltzer, UAMS	10.8 ± 0.8	3
NTH 80	UAMS-690 (Outbreak 1)	M.S. Smeltzer, UAMS	0.5 ± 0.6	1
NTH 81	UAMS-691 (Outbreak 2)	M.S. Smeltzer, UAMS	12.8 ± 1.0	3
NTH 82	UAMS-697	M.S. Smeltzer, UAMS	14.0 ± 0.5	1

*Values represent the mean diameter in millimeters ± the standard deviation of three independent determinations.

^#^Values represent the number of chemiluminescent bands detected when hybridized with a *hysA* specific probe.

**^§^**American Type Culture Collection (ATCC), Manassas, Va.

^**T**^Type strain

**^¶^**Toxic shock syndrome.

^†^Network for Antimicrobial Resistant *Staphylococcus aureus*.

^¦^University of Arkansas for Medical Sciences, Little Rock, Ark.

**^!^**University of Nebraska Medical Center, Omaha, Neb.
